# ‘Sometimes I feel like the only physio in the whole wide world, so alone’

**DOI:** 10.4102/sajp.v81i1.2150

**Published:** 2025-06-09

**Authors:** Brett J. Mason, Romy Parker, Martha Geiger

**Affiliations:** 1 Chronic Pain Management Clinic, Department of Anaesthesia and Perioperative Medicine, Faculty of Health Sciences, University of Cape Town, Cape Town, South Africa; 2 Division of Disability and Rehabilitation Studies, Faculty of Medicine and Health Sciences, Stellenbosch University, Cape Town, South Africa

**Keywords:** chronic pain, primary healthcare, South Africa, physiotherapy, pain management, patient-centred care, rehabilitation, qualitative research

## Abstract

**Background:**

High-impact chronic pain (HICP) presents significant challenges within primary healthcare (PHC) settings, particularly in resource-constrained environments such as South Africa. Limited evidence exists regarding physiotherapists’ lived experiences managing this condition in local contexts.

**Objectives:**

This study explored facilitators and barriers to providing adequate care for individuals with HICP, as experienced by physiotherapists working in Western Cape PHC facilities. It is envisaged that the findings may contribute to curriculum development, policy considerations, and clinical practice enhancement.

**Method:**

A descriptive qualitative approach was employed, and semi-structured virtual interviews were conducted with eight purposively selected physiotherapists from Western Cape PHC facilities. Inductive thematic analysis guided data interpretation.

**Results:**

Analysis revealed four interconnected themes: (1) High-impact chronic pain as a complex and disabling burden in PHC settings, (2) person-centred approaches as key facilitators, (3) systemic and personal barriers constraining effective management, and (4) specific needs for improved HICP care. While person-centred approaches were valued, participants struggled with implementation amid resource constraints.

**Conclusion:**

High-impact chronic pain management in South African PHC settings requires attention to interrelated factors of time, support, and knowledge. While physiotherapists recognise effective approaches, implementation challenges necessitate systemic adjustments.

**Clinical implications:**

These insights highlight the necessity for workforce planning reform, professional development in pain management, and enhanced interdisciplinary collaboration to better serve patients with HICP in resource-limited settings.

## Introduction

Chronic pain represents a complex phenomenon extending beyond tissue damage, evolving from an adaptive protective mechanism to a potentially disabling condition when inadequately managed (Ashton-James et al., 2021). The understanding of pain has evolved since Engel’s biopsychosocial model was introduced in 1977, which posits that pain is not merely a physical sensation but a multidimensional experience shaped by biological, psychological, and social influences (Treede et al. 2019). This perspective has been further developed, suggesting that chronic pain can be viewed as a neuroplastic phenomenon shaped by individual learning and lived experiences (Meints & Edwards 2018).

The global prevalence and impact of chronic pain, as a leading cause of disability, position it as a global health crisis and silent epidemic (Rice, Smith & Blyth 2016; Vos et al. 2020). Chronic pain, which is pain persisting beyond the expected healing timeframe of 6–12 weeks, can significantly disrupt multiple dimensions of daily functioning, including work capacity, social engagement, psychological well-being, and self-care capabilities (Treede et al. 2019). When chronic pain disrupts an individual’s ability to perform activities or their participation in life roles, it is termed ‘high-impact chronic pain’ (HICP) (Dahlhamer et al., 2018). This global challenge is reflected in South African healthcare, where chronic pain significantly impacts individuals and the healthcare system (Igumbor et al. 2011; Kamerman et al. 2020).

International evidence identifies chronic pain as one among the most common presenting complaints in primary healthcare (PHC) settings globally (Smith et al. 2019). Locally, a South African survey found that 18% of the population experience chronic pain, with 61% of affected individuals reported as unemployed and merely 23% receiving disability support (Kamerman et al. 2020). Further analysis of South African PHC consultations revealed that pain-related symptoms constituted 16 of the 50 most frequent presentations within PHC facilities (Mash et al. 2012).

The South African healthcare landscape presents distinct challenges when addressing chronic pain. Approximately 83% – 84% of South Africans access healthcare through the public sector, predominantly via PHC facilities (Mayosi & Benatar 2014). However, this sector, serviced by only 30% of the country’s doctors, faces significant challenges. The public sector spent 4.1% of the annual gross domestic product (GDP) on health in 2017, while the private sector, serving 16% – 17% of the population, spent 4.4% (Ngobeni, Breitenbach & Aye 2020). The challenges faced within the under-resourced and overburdened public healthcare system are compounded by its fragmentation (Kautzky & Tollman 2008) and historical socioeconomic inequalities affecting education, employment, housing, and health status (Finn 2022; Mason 2022). These factors exacerbate the poverty-disability link, particularly in rural and peri-urban communities (Magaqa, Ariana & Polack 2021).

The PHC model, implemented in South Africa following the democratic transition in 1994, aimed to address these historical inequities through improved healthcare access and service delivery (Hone et al. 2018; Kautzky & Tollman 2008). Primary healthcare facilities represent potentially optimal environments for chronic pain management in South Africa because they offer accessible, community-based care that can address pain management early, promote continuity of care, and integrate multidisciplinary approaches tailored to local health needs (Ernstzen, Hillier & Louw 2022; Rispel et al. 2016). Physiotherapists positioned within this system play a potentially pivotal role in addressing chronic pain (Meroni et al. 2021; Morris et al. 2021), with their evolving understanding of pain science expanding their practice scope and potential impact on HICP management (Parker & Madden 2020).

Despite physiotherapists’ unique position, HICP remains a substantial burden within South African PHC settings (Kamerman et al. 2020; Vos et al. 2020). While international literature documents various dimensions of chronic pain management, a significant knowledge gap exists regarding physiotherapists’ lived experiences when managing HICP within South African PHC contexts. Following the comprehensive literature review, no studies were identified that specifically explored physiotherapists’ perceptions of contextual, subjective, or culturally specific factors influencing HICP care in South African PHC.

Understanding these experiences is essential to address gaps in knowledge and inform practice improvements that could benefit the substantial population affected by HICP. The impact of chronic pain on quality of life, workforce participation, and healthcare resource utilisation creates an imperative to understand better and improve management approaches. Therefore, this study aimed to explore the barriers and facilitators to providing effective care for individuals with HICP, as experienced by physiotherapists working in Western Cape PHC settings. It is envisaged that these insights will contribute to enhancing curricula, informing policy development, and improving clinical practice within the South African PHC system.

## Research methods and design

### Study design

This research employed a qualitative descriptive approach with a post-positivist and subjectivist epistemology. This approach facilitated deep engagement with contextual factors through collaborative interaction with participants, allowing rich insights into physiotherapists’ lived experiences.

### Setting

The study was conducted in the Western Cape province of South Africa, which comprises one urban district (City of Cape Town) and five rural districts. This province contains 161 PHC facilities where outpatient rehabilitation services are delivered through various structures, including day hospitals, community health centres, health clinics, and satellite or mobile clinics (Mason 2022).

### Study population and sampling strategy

The research targeted qualified physiotherapists registered with the Health Professions Council of South Africa who are working full-time in Western Cape public sector PHC facilities. A purposive sampling strategy was implemented to ensure representation across the province’s six districts and capture diverse experiences.

Inclusion criteria encompassed community service physiotherapists (newly qualified practitioners completing mandatory year-long public service) and experienced clinicians delivering services in PHC settings. Exclusion criteria included physiotherapists not providing regular full-time clinical services, those on sabbatical, or those on extended leave exceeding 3 months.

The researcher requested consent via the appropriate Department of Health research channels from all 161 PHC facilities in the Western Cape, receiving responses from 26 facilities within the established timeframe. From these responses, 14 potential participants were initially identified, with 10 agreeing to participate. No facilities from the West Coast district responded to research invitations, and only one of two physiotherapists from the Central Karoo district consented to participation. Following the exclusion of two pilot study participants (used to refine the interview guide), data from eight participants informed the final analysis.

The analysed sample included three urban physiotherapists and five rural physiotherapists. Experience levels varied substantially: two participants had less than 1 year of practice (community service), three had 1–5 years of experience, two had 5–10 years of experience, and one had practised for 13 years. Only one participant reported formal pain management training, consisting of a 1-day workshop and a 6-week programme focused on group interventions for chronic pain.

### Data collection

Data were gathered through in-depth, semi-structured virtual interviews conducted between August and October 2022 using the Microsoft Teams platform. The interview guide, refined through pilot testing, explored participants’ understanding of HICP, their perceived role in managing affected individuals, barriers and facilitators to effective care, and perceived needs for practice improvement. Interviews lasted 30–60 min and were audio and video recorded with explicit participant consent.

The lead investigator, a native English speaker, conducted all interviews. While participants represented diverse linguistic backgrounds, all demonstrated English proficiency sufficient for detailed discussion without interpretation needs. Translators were offered to all participants, all of whom declined. Any potential language limitations were addressed through clarification questions and member checking during interviews.

### Data analysis

The research employed inductive reflexive thematic analysis following the six-step framework described by Braun et al. (2019). This process involved (1) gaining familiarity with interview content through repeated reading and initial noting, (2) generating preliminary codes systematically across the dataset, (3) identifying potential thematic patterns, (4) reviewing themes for coherence and distinctiveness, (5) defining and naming themes, and (6) producing the analytical report. This iterative approach facilitated deep engagement with participant experiences and ensured thematic representations authentically reflected their accounts.

Data collection and analysis proceeded concurrently, with each interview informing subsequent enquiries. The concept of theoretical sufficiency, rather than saturation, guided sampling decisions. As described by Dey (1999) and Nelson (2017), theoretical sufficiency emphasises the richness, depth, and diversity of data rather than mere quantity, and this was achieved when analysis revealed no emergent themes.

### Researcher positionality and reflexivity

The primary investigator’s position as a male physiotherapist with Western Cape PHC experience, pursuing postgraduate education while working in a rural PHC setting, provided advantages and potential limitations. This insider perspective enhanced rapport and understanding of context-specific challenges but necessitated careful attention to preventing personal experiences from unduly influencing data collection and analysis.

Several strategies were employed to maintain rigour despite this dual positioning. Firstly, interview questions underwent pilot testing and supervisor review to ensure neutrality. Secondly, during interviews, the investigator consciously maintained an open stance, avoiding sharing personal experiences or leading participants towards particular responses. Thirdly, thematic analysis involved collaboration with supervisors who provided external perspectives challenging potential assumptions. Finally, a reflexive journal documented potential biases and their influence on methodological decisions throughout the research process.

### Transferability

Detailed descriptions of the study context, participant characteristics, and research processes were provided to enhance transferability. While the Western Cape setting has unique elements, it shares substantial characteristics with other South African provinces’ PHC systems, including resource constraints, rural-urban disparities, and structural healthcare arrangements. The inclusion of participants from diverse settings (rural or urban), varying experience levels, and different facility types supports the potential transferability of findings. Detailed methodological documentation enables readers to assess applicability to their specific contexts. While focused on the Western Cape, the identified themes likely reflect challenges and facilitators relevant to similar resource-constrained PHC settings across South Africa and potentially other low- to middle-income countries.

### Ethical considerations

This study received approval from the Stellenbosch University Human Research Ethics Committee (reference number: S21/11/222) and the South African Department of Health (reference number: WC_202201_015). Written informed consent was obtained digitally from all participants before interviews, with all consent documentation securely stored separately from research data. Confidentiality protections included secure data storage, use of pseudonyms in all research documents, and confidentiality agreements with transcription services.

The research adhered to principles outlined in the Declaration of Helsinki (World Medical Association 2013), South African Guidelines for Good Clinical Practice (Department of Health 2020), Medical Research Council Ethical Guidelines for Research (SAMRC 2002), and Department of Health Ethics in Health Research: Principles, Processes and Studies (2015).

## Results

Four key themes emerged from the analysis of participant interviews, reflecting the complex nature of managing HICP in PHC settings.

### Theme 1: High-impact chronic pain: A complex and disabling burden in primary healthcare settings

The first theme from the analysis highlights the multifaceted nature of HICP and its significant impact on physiotherapists’ workload and clinical practice in PHC settings. Participants described HICP as a condition that substantially adds to their workload and challenges their clinical skills. Many expressed feelings of being overwhelmed and isolated:

‘Sometimes I feel like I’m the only physio in the whole wide world. So alone.’ (Participant 5, 2 years experience, rural)

‘But sometimes, as a therapist, it can be overwhelming because you have tried everything with these patients, and they always come back.’ (Participant 5, 2 years experience, rural)

The participants highlighted the complexity of HICP, emphasising psychosocial factors and diagnostic challenges:

‘There are psychosocial factors as well … lots of stress and seeing patients coming from a low socioeconomic environment where the social malice, low employment opportunities, poverty, poor household situations, and the family dynamics in terms of drugs and domestic abuse.’ (Participant 4, 13 years experience, urban)

‘We treat, and we see, okay, there’s no biomechanical issue, there’s nothing on the X-rays. Nothing, nothing, nothing … I will ask them … Do you have any stress? And then they start to pack out.’ (Participant 8, 1 year experience, rural)

They further acknowledged that because of the PHC setting, a different approach is required:

‘Now I’m in a primary health facility, and it’s a completely different way of thinking and managing … [*compared to secondary/tertiary setting*] … now I see there is a big need for me as a professional to understand this concept better to improve my understanding of pain to assist these patients better.’ (Participant 3, 6 years experience, rural)

### Theme 2: Perceived facilitators: Person-centred approaches and strategies

The second theme focused on the approaches and strategies that participants identified as facilitating effective care for individuals with HICP. Participants consistently highlighted collaborative, person-centred approaches as key facilitators:

‘When we do get that team approach, and the patient is compliant. They want to get better. They’re all in. Those patients are the ones that flourish.’ (Participant 1, 3 years experience, urban)

‘We do whatever we can do … we have to see what works for you, what doesn’t work for you. Cause no patient is a textbook patient.’ (Participant 8, 1 year experience, rural)

Patient education and empowerment were seen as crucial:

‘We don’t necessarily have to satisfy our patients with immediate symptomatic relief, but … to empower our patients through education and promotion through self-care management.’ (Participant 4, 13 years experience, urban)

‘I feel like the education plays a big role in understanding where the pain is coming from and why it is there … both physically and from a mental aspect as well.’ (Participant 1, 3 years experience, urban)

The role of manual therapy was debated, with contrasting views from participants (with different experience levels):

‘They come in once a month, and they get touched. They get hands-on therapy … I think they feel the deep intensity of care.’ (Participant 6, 1 year experience, rural)

‘I’ve found oftentimes not putting my hands on the patient to be the most beneficial thing.’ (Participant 7, 5 years experience, rural)

Group therapy and peer support were highlighted as beneficial:

‘They receive encouragement from others when they see others have been in the same boat … those who have successfully overcome their pain can inspire other individuals … this is not the end of it … there is hope.’ (Participant 4, 13 years experience, urban)

### Theme 3: Systemic and personal barriers constraining effective management

The third theme encapsulates the various barriers that participants identified as limiting their ability to provide effective care for individuals with HICP. A significant barrier mentioned by multiple participants was the lack of time:

‘By the time I get to see most of my patients … it becomes chronic, it’s no longer acute injuries, and out of those we tend to see a lot of high impact chronic pain conditions.’ (Participant 7, 5 years experience, rural)

‘I go there [*the clinics*] once a month, which is not sufficient … and we now go there, there’s like 16 patients that you see, which is quite impossible.’ (Participant 3, 6 years experience, rural)

‘When there’s only one physio … responsible for five clinics, they don’t get to be followed up as frequently or regularly as they might need or want to.’ (Participant 5, 2 years experience, rural)

‘So now I see the patient, but I’m fully booked for a month and a half in advance already. So now this patient that actually needs more attention and more care in the beginning have to wait for that long to be seen again.’ (Participant 6, 1 year experience, rural)

System factors, including lack of support and collaboration, were problematic:

‘I’m the only one who sees them … no teamwork, only me, myself and I.’ (Participant 5, 2 years experience, rural)

‘It’s overloaded because they [*other healthcare professionals*] don’t do certain stuff that they need to do. They’re just like … “we’re just gonna refer down to the physio”.’ (Participant 8, 1 year experience, rural)

‘The mental health component that’s sometimes not always addressed in primary healthcare … doctors and nurses just give them pain medication, pain medication, pain medication. And they come back and they come back and they come back.’ (Participant 3, 6 years experience, rural)

Participants also noted patient-related factors as barriers, including lack of compliance (adherence) and motivation with therapy and home exercise, poor insight into their health and conditions, and unrealistic expectations (Mason 2022):

‘It all depends on the patient’s motivation to get better … and also the compliancy with listening to the advice the physios offer in terms of education and strategies.’ (Participant 4, 13 years experience, urban)

‘They often don’t understand how their condition is contributing to their pain.’ (Participant 5, 2 years experience, rural)

‘They do expect that after session one, they are healed- miracle, whatever.’ (Participant 1, 3 years experience, urban)

Access to services was highlighted as a barrier:

‘I can only go out to three [*clinics*] and the other patients has to come to me … the other clinics I don’t go to. The patients must come to me at the hospital because of the high inpatient load I have.’ (Participant 3, 6 years experience, rural)

The participant’s own knowledge gaps were also acknowledged as barriers:

‘If you don’t understand something completely, how do you explain it to someone else?’ (Participant 3, 6 years experience, rural)

‘Communicating to the patients. I feel like it’s such a complex thing. I don’t personally understand it [*HICP*] … so how do I simplify it to explain it to them.’ (Participant 3, 6 years experience, rural)

### Theme 4: Perceived needs for improved high-impact chronic pain care

The final theme that emerged from the analysis relates to the needs identified by participants for improving HICP care. Participants consistently expressed a need for more time and staff:

‘What I need? Another therapist.’ (Participant 3, 6 years experience, rural)

‘People have to wait mainly two months to be seen … there’s definitely a need for another physio in this district.’ (Participant 6, 1 year experience, rural)

‘In an ideal world … I would have a minimum of three physios for the two subdistricts.’ (Participant 7, 5 years experience, rural)

They highlighted the need for better support and management:

‘It would be lovely to have a rehab manager or an allied manager just to have our backs because in the clinics, being a sister/nurse as a manager, they tend to … show more favouritism towards their profession.’ (Participant 2, 3 years experience, urban)

‘If we maybe had a separate service, that could offer that [*chronic pain groups*], that we could refer patients to not necessarily us run it ourselves … it could kind of lessen the load on the clinics because those patients can be treated somewhere else … we’re not ignoring them. We’re just giving them another opportunity.’ (Participant 4, 13 years experience, urban)

Participants emphasised the need for more knowledge, training, and context-specific resources:

‘I need to improve my knowledge about chronic pain and then the different management strategies there are of these patients.’ (Participant 3, 6 years experience, rural)

‘It starts with me … I have to be educated in order to treat my patients.’ (Participant 6, 1 year experience, rural)

‘Of course, it would be nice if there’s like a … guideline, a semi-guideline and … culturally adapted outcome measurement tools that’s applicable to your facility and your home language.’ (Participant 3, 6 years experience, rural)

These themes provide a comprehensive insight into the complex nature of managing HICP in PHC settings, highlighting the multifaceted challenges faced by physiotherapists, the strategies they find effective, and their perceived needs for improvement. The findings underscore the interplay between systemic issues, patient factors, and physiotherapist capabilities in the context of HICP management in South African PHC settings.

## Discussion

The qualitative descriptive methodology employed in this study revealed four key themes that highlight the complex nature of managing HICP in South African PHC settings. These themes underscore the interrelationships between time, support, and knowledge, which directly translate to the identified needs of physiotherapists working in this context. Each theme provides valuable insights into the on-the-ground challenges and opportunities for improving HICP care in PHC settings.

### Theme 1: High-impact chronic pain: A complex and disabling burden in primary healthcare settings

Participants in our study portrayed HICP cases as particularly demanding, requiring significant time investment and specialised attention amid resource constraints. For instance, a participant noted that approximately 80% – 85% of their clinical workload consisted of individuals with chronic pain (Participant 1, 3 years experience, urban). The emotional impact of this burden was powerfully captured in Participant 5’s statement (2 years experience, rural) about feeling like ‘the only physio in the whole wide world, so alone’. This isolation, particularly affecting younger and less experienced clinicians, aligns with broader evidence that physiotherapists working in South African PHC settings encounter a diverse array of conditions affecting the majority of the population (Ned, Cloete & Mji 2017). These contexts necessitate that they assume generalist roles, often being the sole physiotherapist in extensive areas with limited resources and support (Ned et al. 2017), reflecting how working in PHC settings requires managing complex conditions with limited support structures and professional collaboration (Campo, Weiser & Koenig 2009).

Beyond the challenges of isolation, the complexity of HICP in PHC settings was emphasised through participants’ descriptions of multifaceted patient presentations involving psychosocial factors like ‘stress … low socioeconomic environment … poverty, poor household situations, and family dynamics’ (Participant 4, 13 years experience, urban). Their observations of cases where ‘there’s no biomechanical issue … nothing on the X-rays’ (Participant 8, 1 year experience, rural) highlight the challenge of managing conditions that extend beyond simple physical presentations. This complexity adds another layer to the physiotherapists’ burden, particularly when working in isolation, and necessitates a unique approach to care delivery. The disconnect between traditional biomedical training and the complex biopsychosocial nature of HICP suggests a need for specialised pain management education that equips physiotherapists to better understand and address these multifaceted presentations (Holopainen et al. 2020; Ng et al. 2021).

The distinct nature of managing HICP in PHC settings compared to secondary and/or tertiary care was clearly articulated by participants, with (Participant 3, 6 years experience, rural) noting it requires ‘a completely different way of thinking and managing’. This aligns with core PHC principles of accessibility, community participation, prevention and health promotion, integration, intersectoral collaboration, appropriate technology, adequate health workforce, and quality of care (Hone et al. 2018). However, participant responses revealed discrepancies between these theoretical principles and their practical implementation (Rispel, 2016; Visagie & Schneider 2014), suggesting that managing HICP effectively in PHC settings requires not just clinical expertise but also the ability to adapt theoretical knowledge to resource-limited contexts while addressing broader social determinants of health (Stanos et al. 2016).

### Theme 2: Perceived facilitators: Person-centred approaches and strategies

Participants identified that effective care for individuals with HICP is facilitated through collaborative, person-centred approaches that emphasise therapeutic alliance and continuity of care. The importance of creating a therapeutic space where patients feel heard aligns with established biopsychosocial models of care (Dobscha et al., 2009; Peppin et al. 2015). As highlighted by (Participant 8, 1 year experience, rural)’s quote about there being ‘no textbook patient’, participants recognised that each individual requires a unique approach. This therapeutic alliance, characterised by empathy and non-judgement, is crucial for fostering positive outcomes in chronic pain management (Kinney et al., 2020; Wilson et al. 2017). The participants’ emphasis on communication, sensitivity, and patient-perceived care reflects their understanding of the benefits of person-centred care in PHC settings (Morera-Balaguer et al. 2019).

Building on these therapeutic relationships, education and empowerment emerged as crucial strategies, with participants highlighting the shift from passive interventions to active self-management approaches. This strategy of facilitating experiential learning and adaptation aligns with person-centred care principles in PHC that aim to enhance individuals’ ability to achieve their personalised goals through continuous support (Hammond, Stenner & Palmer 2022). The provision of education and individualised exercise programmes were highlighted as a strategy that promotes insight into patients’ conditions. This experiential and collaborative learning strategy moves away from passive, temporarily relieving, and resource-intensive modalities towards a more function-orientated, personalised, and activity-focused model that enhances patients’ ability to learn, adapt, and engage better in daily activities (Girbés et al. 2015; Mason 2022; Morera-Balaguer et al. 2019).

The implementation of treatment strategies revealed interesting contrasts, particularly regarding manual therapy. While a less experienced clinician noted that hands-on therapy fostered a sense of ‘deep intensity of care’ (Participant 6, 1 year experience, rural), a more experienced practitioner found that ‘not putting hands on the patient’ was often most beneficial (Participant 7, 5 years experience, rural). The approach of using manual therapy or hands-on techniques for managing persistent pain conditions continues to generate debate among practitioners (Bialosky et al. 2017). A more nuanced perspective suggests that successful outcomes may not depend primarily on whether manual therapy is employed or avoided during treatment. Instead, benefits might stem largely from contextual factors such as the psychological impact of treatment expectations and the quality of practitioner-patient relationships established during clinical encounters (Bishop et al. 2015; Girbés et al. 2015; Mason 2022).

Beyond individual treatment approaches, group therapy and peer support were identified as valuable strategies that foster community and motivation while potentially reducing clinician burden. Group therapy and patient-led peer support groups for chronic pain have demonstrated positive results for several chronic conditions in South Africa, including chronic pain (Barnes et al., 2019; Jackson et al. 2021; Parker & Madden 2020; Saw et al., 2016). In fact, (Participant 4, 13 years experience, urban)’s observation about patients receiving ‘encouragement from others’ and finding inspiration through peer success stories emphasises these benefits. However, implementing such approaches in PHC settings requires strategic planning, knowledge, and time for implementation, maintenance, and evaluation (Gilmore et al. 2017). Despite these implementation challenges in resource-constrained settings, participants recognised the value of peer support in creating hope and fostering self-management skills among individuals with HICP.

### Theme 3: Systemic and personal barriers constraining effective management

Time constraints emerged as a pervasive barrier, with participants describing overwhelming caseloads and insufficient frequency of treatment sessions. In this regard, (Participant 7, 5 years experience, rural)’s observation that ‘by the time I get to see most of my patients… it becomes chronic’ highlights how limited availability can transform acute conditions into chronic problems. This issue is particularly acute in rural settings where single practitioners must service multiple facilities, with participants reporting up to 3-month waiting periods and responsibilities across multiple clinics. These time constraints have been well documented in both local (Major-Helsloot et al. 2014) and international studies (Condon et al., 2016; Scurlock-Evans et al., 2014), directly contradicting PHC principles of accessibility and timely intervention. These time constraints, while significant on their own, are further exacerbated by systemic barriers within the PHC framework.

System-level barriers manifested through inadequate support structures and poor interdisciplinary collaboration. Participants described feeling isolated (‘no teamwork, only me, myself and I’ – (Participant 5, 2 years experience, rural)) and unsupported by other healthcare professionals who often inappropriately referred patients without proper screening or management. This breakdown in collaborative care directly contradicts evidence supporting interdisciplinary approaches for effective chronic pain management (Stanos et al. 2016). The issue is compounded by management structures that lack rehabilitation expertise, leading to feelings of being ‘overlooked’ and decisions being made without understanding the role and capabilities of physiotherapists. The diverse distribution of services within PHC and the complexity of its scope (Hone et al., 2018), coupled with insufficient interdisciplinary collaboration, which is deemed essential for pain management in community-based settings (Stanos et al. 2016), substantially restricts physiotherapists’ capacity to deliver effective treatment interventions. These systemic challenges inevitably influence patient engagement and outcomes in treatment.

Within this challenging system, patient-related factors reflected broader healthcare engagement challenges, particularly regarding agency and motivation. The evolution from using ‘compliance’ to ‘adherence’ in contemporary healthcare literature (Settineri et al., 2019) highlights the need to move away from paternalistic approaches. Participants’ observations about patients’ limited insight and unrealistic expectations (‘they do expect that after session one, they are healed’ – [Participant 1, 3 years experience, urban]) suggest a need for better patient education and expectation management. These challenges are compounded by socioeconomic factors affecting treatment adherence, reflecting broader healthcare access inequities in South Africa (Binagwaho & Ghebreyesus 2019). Recent research has uncovered various factors that may influence treatment adherence, such as the therapeutic relationship and prescription of individualised home-based programmes (Mason 2022; McLeod et al. 2022). The challenges of patient engagement are further complicated by significant access barriers throughout the healthcare system.

Access barriers highlighted significant gaps in service delivery, particularly in rural areas. (Participant 3, 6 years experience, rural)’s description of only being able to service 3 out of 10 clinics because of inpatient loads exemplifies how resource constraints affect service accessibility. Access has five dimensions (availability, accessibility, affordability, adequacy, and acceptability) according to the ACCESS framework (Mutwali & Ross, 2019; Scheffler et al., 2015). Participants acknowledged barriers related to these dimensions, creating a cycle where limited access leads to poorer outcomes and increased disability. This directly contradicts PHC principles of equity and universal accessibility, as well as the UN 2030 Sustainable Development Goals for improved health and well-being (Binagwaho & Ghebreyesus 2019; Mutwali & Ross 2019). Beyond these structural barriers, physiotherapists’ own knowledge and capacity emerged as a significant concern.

Knowledge barriers were acknowledged at both personal and systemic levels. The candid admission ‘If you don’t understand something completely, how do you explain it to someone else?’ (Participant 3, 6 years experience, rural) reflects the challenge of translating complex pain science into practical patient education. As the trend for most physiotherapists’ training has leaned more towards the biomedical model of care (Girbés et al. 2015), many may find difficulty with recognising and responding to psychological and social dimensions of health, or to integrate the biopsychosocial elements confidently into their clinical practice (Holopainen et al. 2020). A systematic review identified several obstacles preventing clinicians from embracing whole-person approaches to care, including individual practitioner characteristics, gaps in professional preparation, misinterpretations of best practice recommendations, perceptions of patient needs, and practical time constraints during individual consultations (Ng et al. 2021). The challenge is particularly acute in PHC settings where practitioners must maintain generalist knowledge while also developing expertise in complex conditions like HICP (Holopainen et al. 2020).

The interconnected nature of these diverse obstacles forms an intricate network of difficulties that considerably hampers therapists’ capacity to deliver quality treatment to patients experiencing chronic pain within PHC facilities. The interplay between systemic, personal, and patient-related factors suggests that addressing any single barrier in isolation may be insufficient; rather, a comprehensive approach addressing time, support, and knowledge factors simultaneously may be necessary for meaningful improvement in care delivery.

### Theme 4: Perceived needs for improved high-impact chronic pain care

To facilitate maximal practical application and in the hope that findings would meaningfully inform educational frameworks, operational guidelines, and clinical approaches, participants were explicitly asked what resources they required to enhance outcomes when treating patients with HICP in PHC settings. Their responses consistently highlighted needs relating to time, knowledge, and support, with a particularly strong emphasis on the need for additional physiotherapy posts to address overwhelming caseloads.

The overwhelming need for more physiotherapists to create adequate time for effective care was a resounding theme. Although research strongly validates the necessity and value of rehabilitation programmes within PHC frameworks (Bim et al. 2021; Guilcher, 2018; Jesus et al. 2016; Rodés et al. 2021), the World Health Organization has yet to establish standard guidelines for appropriate therapist-to-community ratios (Cieza et al., 2020; Rodés et al. 2021). This crucial oversight in healthcare resource planning continues despite research indicating that rehabilitation services would benefit approximately one-third of individuals during illness, with musculoskeletal disorders representing the majority of these cases (Cieza et al., 2020). The absence of such guidelines particularly impacts HICP management, where regular intervention and adequate time per patient are crucial for effective outcomes.

Beyond workforce needs, participants expressed a clear need for improved support structures. The provision of rehabilitation-proficient management and administrative support could allow clinicians more focused clinical time, with evidence showing that appropriate leadership and management significantly impacts service delivery and staff retention in healthcare settings (Jesus et al. 2016; Morris et al. 2021; Scheffler et al. 2015). Some participants suggested that external services, such as chronic pain groups led by third parties or university students, could help alleviate their workload and enable more focused interventions for individuals with HICP. (Participant 4, 13 years experience, urban)’s suggestion of a ‘separate service’ for chronic pain groups reflects evidence supporting the effectiveness of externally provided group interventions in South African contexts (Barnes et al. 2019; Parker & Madden 2020). While such external services could potentially reduce clinician burden while maintaining care quality, their implementation would require careful integration with existing PHC services and clear referral pathways (Guilcher, 2018; Jesus et al. 2016).

Knowledge-related needs centred around the necessity for chronic pain training and culturally informed guidelines. The findings of this study reflect patterns observed in additional research within the South African healthcare context, which has documented several barriers limiting clinicians from adopting standardised treatment protocols, including a lack of knowledge of existing guidelines, perceived disconnection or malalignment between recommendations and local realities, and inadequate resources or institutional backing needed for effective implementation (Dizon et al., 2017; Stander, Grimmer & Brink 2020). While evidence-based guidelines for chronic pain management exist, participants’ responses suggest a significant gap between available knowledge and practical implementation. This gap appears to stem from challenges in knowledge translation, contextual adaptation, and practical application within resource-constrained settings (Ernstzen et al. 2022; Stander et al. 2020). The apparent disconnect between available research evidence and clinical practice highlights not only the need for improved knowledge translation strategies in PHC settings but also the importance of addressing implementation barriers through context-specific support and training.

These identified needs – time through increased staffing, improved support structures, and enhanced knowledge resources – are inherently interconnected. The absence of workforce guidelines particularly impacts the ability to advocate for additional posts, which in turn affects time available for implementing evidence-based practice and accessing training opportunities. Addressing these needs requires a comprehensive approach that considers both immediate practical solutions and longer-term structural changes to the healthcare system, with particular attention to establishing clear workforce planning guidelines for physiotherapy services in PHC settings.

### Recommendations

Based on the study findings, we recommend assessing PHC physiotherapists’ understanding of HICP and its management strategies to inform targeted training programmes and curriculum development. Increasing support through additional personnel and resources is crucial. Chronic pain training should be incorporated into undergraduate curricula and made accessible to all PHC medical professionals. Physiotherapists should be equipped with skills for innovative practice and networking. Further research, both qualitative and quantitative, is needed to explore HICP management in PHC settings more comprehensively and to determine an ideal PHC physiotherapist-to-workforce population ratio. These recommendations aim to enhance the capacity of PHC facilities to manage HICP effectively and improve patient outcomes.

## Conclusion

High-impact chronic pain presents a complex and disabling burden for physiotherapists in South African PHC settings. This study reveals that while physiotherapists recognise the value of person-centred and collaborative approaches in enhancing treatment effectiveness, they face significant challenges related to time, support, and knowledge that hinder optimal care delivery. These challenges are particularly acute given the absence of clear workforce planning guidelines and the complex nature of HICP management in resource-constrained settings.

Addressing these barriers requires a comprehensive strategy that considers both immediate practical solutions and longer-term structural changes to the healthcare system. Key recommendations emerging from this research include establishing clear workforce-to-population ratios, increasing the number of physiotherapists in PHC settings, appointing rehabilitation-proficient managers, and providing context-specific pain management training. The implementation of these recommendations must consider the inherent connection between time, support, and knowledge factors identified in this study.

This research contributes a nuanced understanding of HICP management in South African PHC from physiotherapists’ perspectives, highlighting the discrepancy between PHC principles and their practical implementation. It underscores the urgent need for systemic changes to support healthcare professionals in delivering effective, person-centred care. Beyond simply increasing resources, successful implementation of these recommendations requires addressing the complex interplay between workforce planning, support structures, and knowledge translation to meaningfully improve outcomes for individuals with HICP in PHC settings.

## References

[CIT0001] Ashton-James, C., Anderson, S.R., Mackey, S.C. & Darnall, B.D., 2021, ‘Beyond pain, distress, and disability: The importance of social outcomes in pain management research and practice’, *Pain* 162(11), 2621–2780. 34252908 10.1097/j.pain.0000000000002404 PMC8742845

[CIT0002] Barnes, R., Jelsma, J. & Parker, R., 2019, ‘Improvements in health-related quality of life and function in middle-aged women with chronic diseases of lifestyle after participating in a non-pharmacological intervention’, *African Journal of Disability* 8, 1–14. 10.4102/ajod.v8i0.428 PMC6424002 30899683

[CIT0003] Bialosky, J.E., Bishop, M.D. & Penza, C.W., 2017, ‘Placebo mechanisms of manual therapy: A sheep in Wolf’s clothing?’, *Journal of Orthopaedic and Sports Physical Therapy* 47(5), 301–304. 10.2519/jospt.2017.0604 28459190

[CIT0004] Bim, C.R., Carvalho, B.G.D., Trelha, C.S., Ribeiro, K.S.Q.S., Baduy, R.S. & González, A.D., 2021, ‘Physiotherapy practices in primary health care’, *Fisioterapia em Movimento* 34, 1–10. 10.1590/fm.2021.34109

[CIT0005] Binagwaho, A. & Ghebreyesus, T.A., 2019, ‘Primary healthcare is cornerstone of universal health coverage’, *BMJ* 365, l2391. 10.1136/bmj.l2391 31160322

[CIT0006] Bishop, M.D., Torres-Cueco, R., Gay, C.W., Lluch-Girbés E., Beneciuk, J.M. & Bialosky, J.E., 2015, ‘What effect can manual therapy have on a patient’s pain experience?’, *Pain Management* 5(6), 455–464. 10.2217/pmt.15.39 26401979 PMC4976880

[CIT0007] Braun, V., Clarke, V., Hayfield, N. & Terry, G., 2019, ‘Thematic analysis’, in P. Liamputtong (ed.), *Handbook of research methods in health social sciences*, 2nd edn., pp. 843–860, Springer, Singapore.

[CIT0008] Campo, M., Weiser, S. & Koenig, K., 2009, ‘Job strain in physical therapists’, *Physical Therapy* 89(9), 946–956. 10.2522/ptj.20080322 19608632 PMC2737052

[CIT0009] Cieza, A., Causey, K., Kamenov, K., Hanson, S.W., Chatterji, S. & Vos, T., 2020, ‘Global estimates of the need for rehabilitation based on the Global Burden of Disease study 2019: A systematic analysis for the Global Burden of Disease’, *The Lancet* 396(10267), 2006–2017. 10.1016/S0140-6736(20)32340-0 PMC7811204 33275908

[CIT0010] Dahlhamer, J., Lucas, J., Zelaya, C., Nahin, R., Mackey, S., DeBar, L. et al., 2018, ‘Prevalence of chronic pain and high-impact chronic pain among adults – United States, 2016’, *Morbidity and Mortality Weekly Report* 67(36), 1001–1006. 10.15585/mmwr.mm6736a2 30212442 PMC6146950

[CIT0011] Department of Health, 2015, *Ethics in health research principles, processes and structures*, viewed 28 August 2022, from https://knowledgehub.health.gov.za/elibrary/ethics-health-research-principles-processes-and-structures.

[CIT0012] Department of Health, 2020, *South African good clinical practice: Clinical trial guidelines*, Department of Health, Republic of South Africa, Pretoria.

[CIT0013] Dey, I., 1999, *Grounding grounded theory: Guidelines for qualitative inquiry*, Academic Press, San Diego, CA.

[CIT0014] Dizon, J.M., Grimmer, K., Louw, Q., Machingaidze, S., Parker, H. & Pillen, H., 2017, ‘Barriers and enablers for the development and implementation of allied health clinical practice guidelines in South African primary healthcare settings: A qualitative study’, *Health Research Policy and Systems* 15(1), 1–13. 10.1186/s12961-017-0243-3 28915890 PMC5603069

[CIT0015] Dobscha, S.K., Corson, K., Perrin, N.A., Hanson, G.C., Leibowitz, R.Q., Doak, M.N. et al., 2009, ‘Collaborative care for chronic pain in primary care: A cluster randomized trial’, *JAMA* 301(12), 1242–1252. 10.1001/jama.2009.377 19318652

[CIT0016] Engel, G.L., 1977, ‘The need for a new medical model: A challenge for biomedicine’, *Science* 196(4286), 129–136. 10.1126/science.847460 847460

[CIT0017] Ernstzen, D.V., Hillier, S.L. & Louw, Q.A., 2022, ‘Synthesis of clinical practice guideline recommendations for the primary health care of chronic musculoskeletal pain’, *Journal of Evaluation in Clinical Practice* 28(3), 454–467. 10.1111/jep.13644 34913219

[CIT0018] Finn, B., 2022, ‘Pandemic urbanization: How South Africa’s history of labor and disease control creates its current disparities’, *Journal of Urban Affairs* 2022, 1–14.

[CIT0019] Gilmore, B., MacLachlan, M., McVeigh, J., McClean, C., Carr, S., Duttine, A. et al., 2017, ‘A study of human resource competencies required to implement community rehabilitation in less resourced settings’, *Human Resources for Health* 15(1), 1–14. 10.1186/s12960-017-0240-1 28938909 PMC5610467

[CIT0020] Girbés, E.L., Meeus, M., Baert, I. & Nijs, J., 2015, ‘Balancing “hands-on” with “hands-off” physical therapy interventions for the treatment of central sensitization pain in osteoarthritis’, *Manual Therapy* 20(2), 349–352. 10.1016/j.math.2014.07.017 25169787

[CIT0021] Guilcher, S.J., 2018, ‘The value of physiotherapists in primary health care clinics: Optimizing (self-) management supports for persons with complex health and social needs’, *Physiotherapy Canada* 70(1), 1–2. 10.3138/ptc.70.1.gee 29434412 PMC5802961

[CIT0022] Hammond, R., Stenner, R. & Palmer, S., 2022, ‘What matters most: A qualitative study of person-centered physiotherapy practice in community rehabilitation’, *Physiotherapy Theory and Practice* 38(9), 1207–1218. 10.1080/09593985.2020.1825577 33044879

[CIT0023] Holopainen, R., Simpson, P., Piirainen, A., Karppinen, J., Schütze, R., Smith, A. et al., 2020, ‘Physiotherapists’ perceptions of learning and implementing a biopsychosocial intervention to treat musculoskeletal pain conditions’, *Pain* 161(6), 1150–1168. 10.1097/j.pain.0000000000001809 31977935

[CIT0024] Hone, T., Macinko, J. & Millett, C., 2018, ‘Revisiting Alma-Ata: What is the role of primary health care in achieving the Sustainable Development Goals?’, *Lancet* 392(10156), 1461–1472. 10.1016/S0140-6736(18)31829-4 30343860

[CIT0025] Igumbor, E.U., Puoane, T.R., Gansky, S.A. & Plesh, O., 2011, ‘Chronic pain in the community: A survey in a township in Mthatha, Eastern Cape, South Africa’, *Southern African Journal of Anaesthesia and Analgesia* 17(5), 329–337. 10.1080/22201173.2011.10872801

[CIT0026] Jackson, T., Thomas, S., Stabile, V., Han, X., Shotwell, M. & McQueen, K.A., 2016, ‘A systematic review and meta-analysis of the global burden of chronic pain without clear etiology in low-and middle-income countries: Trends in heterogeneous data’, *Anesthesia & Analgesia* 123(3), 739–748. 10.1213/ANE.0000000000001389 27537761

[CIT0027] Jesus, T.S., Koh, G., Landry, M., Ong, P.H., Lopes, A.M., Green, P.L. et al., 2016, ‘Finding the “right-size” physical therapy workforce: International perspective across 4 countries’, *Physical Therapy* 96(10), 1597–1609. 10.2522/ptj.20160014 27149960

[CIT0028] Kamerman, P.R., Bradshaw, D., Laubscher, R., Pillay-van Wyk, V., Gray, G.E., Mitchell, D. et al., 2020, ‘Almost 1 in 5 South African adults have chronic pain: A prevalence study conducted in a large nationally representative sample’, *Pain* 161(7), 1629–1635. 10.1097/j.pain.0000000000001844 32102020

[CIT0029] Kautzky, K. & Tollman, S., 2008, ‘A perspective on primary health care in South Africa: Primary health care: In context’, *South African Health Review* 2008(1), 17–30.

[CIT0030] Kinney, M., Seider, J., Beaty, A.F., Coughlin, K., Dyal, M. & Clewley, D., 2020, ‘The impact of therapeutic alliance in physical therapy for chronic musculoskeletal pain: A systematic review of the literature’, *Physiotherapy Theory and Practice* 36(8), 886–898. 10.1080/09593985.2018.1516015 30265840

[CIT0031] Magaqa, Q., Ariana, P. & Polack, S., 2021, ‘Examining the availability and accessibility of rehabilitation services in a rural District of South Africa: A mixed-methods study’, *International Journal of Environmental Research and Public Health* 18(9), 4692. 10.3390/ijerph18094692 33924910 PMC8125304

[CIT0032] Major-Helsloot, M.E., Crous, L.C., Grimmers-Somers, K. & Louw, Q.A., 2014, ‘Management of LBP at primary care level in South Africa: Up to standards?’, *African Health Sciences* 2014(3), 698–706. 10.4314/ahs.v14i3.28 PMC4209660 25352891

[CIT0033] Mash, B., Fairall, L., Adejayan, O., Ikpefan, O., Kumari, J., Mathee, S. et al., 2012, ‘A morbidity survey of South African primary care’, *PLoS One* 7(3), e32358. 10.1371/journal.pone.0032358 22442666 PMC3306367

[CIT0034] Mason, B.J.N., 2022, ‘High-impact chronic pain: Barriers and facilitators identified by Western Cape primary healthcare physiotherapists’, Master of Human Rehabilitation Studies Thesis, Stellenbosch University, viewed from https://scholar.sun.ac.za/server/api/core/bitstreams/36a5099f-2984-4a92-82f6-bcc39e703434/content

[CIT0035] Mayosi, B.M. & Benatar, S.R., 2014, ‘Health and health care in South Africa – 20 years after Mandela’, *New England Journal of Medicine* 371(14), 1344–1353. 10.1056/NEJMsr1405012 25265493

[CIT0036] McLeod, G., Morgan, E., McMillan, S., McCahon, S. & Sanna, N., 2022, ‘Why are patients not doing their prescribed home-based exercises? An updated review of the factors affecting adherence to prescribed home-based exercise in patients with chronic low back pain’, *Home Health Care Management and Practice* 35(2), 114–122. 10.1177/10848223221116143

[CIT0037] Meints, S.M. & Edwards, R.R., 2018, ‘Evaluating psychosocial contributions to chronic pain outcomes’, *Progress in Neuro-Psychopharmacology and Biological Psychiatry* 87, 168–182. 10.1016/j.pnpbp.2018.01.017 29408484 PMC6067990

[CIT0038] Meroni, R., Piscitelli, D., Ravasio, C., Vanti, C., Bertozzi, L., De Vito, G. et al., 2021, ‘Evidence for managing chronic low back pain in primary care: A review of recommendations from high-quality clinical practice guidelines’, *Disability and Rehabilitation* 43(7), 1029–1043. 10.1080/09638288.2019.1645888 31368371

[CIT0039] Morera-Balaguer, J., Botella-Rico, J.M., Catalán-Matamoros, D., Martínez-Segura, O.R., Leal-Clavel, M. & Rodríguez-Nogueira, Ó., 2019, ‘Patients’ experience regarding therapeutic person-centered relationships in physiotherapy services: A qualitative study’, *Physiotherapy Theory and Practice* 37(1), 17–27. 10.1080/09593985.2019.1603258 31002005

[CIT0040] Morris, L.D., Grimmer, K.A., Twizeyemariya, A., Coetzee, M., Leibbrandt, D.C. & Louw, Q.A., 2021, ‘Health system challenges affecting rehabilitation services in South Africa’, *Disability and Rehabilitation* 43(6), 877–883. 10.1080/09638288.2019.1641851 31378096

[CIT0041] Mutwali, R. & Ross, E., 2019, ‘Disparities in physical access and healthcare utilization among adults with and without disabilities in South Africa’, *Disability and Health Journal* 12(1), 35–42. 10.1016/j.dhjo.2018.07.009 30082200

[CIT0042] Ned, L., Cloete, L. & Mji, G., 2017, ‘The experiences and challenges faced by rehabilitation community service therapists within the South African Primary Healthcare health system’, *African Journal of Disability* 6(1), 1–11. 10.4102/ajod.v6i0.311 PMC5645568 29062760

[CIT0043] Nelson, J., 2017, ‘Using conceptual depth criteria: Addressing the challenge of reaching saturation in qualitative research’, *Qualitative Research* 17(5), 554–570. 10.1177/1468794116679873

[CIT0044] Ng, W., Slater, H., Starcevich, C., Wright, A., Mitchell, T. & Beales, D., 2021, ‘Barriers and enablers influencing healthcare professionals’ adoption of a biopsychosocial approach to musculoskeletal pain: A systematic review and qualitative evidence synthesis’, *Pain* 162(8), 2154–2185. 10.1097/j.pain.0000000000002217 33534357

[CIT0045] Ngobeni, V., Breitenbach, M.C. & Aye, G.C., 2020, ‘Technical efficiency of provincial public healthcare in South Africa’, *Cost Effectiveness and Resource Allocation* 18(1), 3. 10.1186/s12962-020-0199-y 32002018 PMC6986147

[CIT0046] Parker, R. & Madden, V., 2020, ‘State of the art: What have the pain sciences brought to physiotherapy?’, *South African Journal of Physiotherapy* 76(1), 1–6. 10.4102/sajp.v76i1.1390 PMC7059532 32161828

[CIT0047] Peppin, J.F., Cheatle, M.D., Kirsh, K.L. & McCarberg, B.H., 2015, ‘The complexity model: A novel approach to improve chronic pain care’, *Pain Medicine* 16(4), 653–666. 10.1111/pme.12621 25752874

[CIT0048] Rice, A.S.C., Smith, B.H. & Blyth, F.M., 2016, ‘Pain and the global burden of disease’, *Pain* 157(4), 791–796. 10.1097/j.pain.0000000000000454 26670465

[CIT0049] Rispel, L.C., 2016, ‘Analysing the progress and fault lines of health sector transformation in South Africa’, *South African Health Review* 2016(1), 17–23.

[CIT0050] Rodés, C.H., Daré, J.V.L., De Araujo, B.C., Graciani, L., João, S.M.A., Germani, A.C.C.G. et al., 2021, ‘The physiotherapy workforce in the Brazilian Unified Health Care System’, *Human Resources for Health* 19(1), 1–11. 10.1186/s12960-021-00642-8 34419076 PMC8379878

[CIT0051] SAMRC, 2002, *Ethical guidelines for research*, South African Medical Research Council, Cape Town.

[CIT0052] Saw, M.M., Kruger-Jakins, T., Edries, N. & Parker, R., 2016, ‘Significant improvements in pain after a six-week physiotherapist-led exercise and education intervention, in patients with osteoarthritis awaiting arthroplasty, in South Africa: A randomised controlled trial’, *BMC Musculoskeletal Disorders* 17(1), 1–14. 10.1186/s12891-016-1088-6 27233479 PMC4884378

[CIT0053] Scheffler, E., Visagie, S. & Schneider, M., 2015, ‘The impact of health service variables on healthcare access in a low resourced urban setting in the Western Cape, South Africa’, *African Journal of Primary Health Care and Family Medicine* 7(1), 1–11. 10.4102/phcfm.v7i1.820 PMC4656938 26245611

[CIT0054] Settineri, S., Frisone, F., Merlo, E.M., Geraci, D. & Martino, G., 2019, ‘Compliance, adherence, concordance, empowerment, and self-management: Five words to manifest a relational maladjustment in diabetes’, *Journal of Multidisciplinary Healthcare* 12, 299. 10.2147/JMDH.S193752 31118655 PMC6499139

[CIT0055] Smith, B.H., Fors, E.A., Korwisi, B., Barke, A., Cameron, P., Colvin, L. et al., 2019, ‘The IASP classification of chronic pain for ICD-11: Applicability in primary care’, *Pain* 160(1), 83–86. 10.1097/j.pain.0000000000001360 30586075

[CIT0056] Stander, J., Grimmer, K. & Brink, Y., 2020, ‘Factors influencing clinical practice guideline uptake by South African physiotherapists: A qualitative investigation of barriers and facilitators’, *Journal of Evaluation in Clinical Practice* 26(3), 728–737. 10.1111/jep.13182 31190423

[CIT0057] Stanos, S., Brodsky, M., Argoff, C., Clauw, D.J., D’Arcy, Y., Donevan, S. et al., 2016, ‘Rethinking chronic pain in a primary care setting’, *Postgraduate Medicine* 128(5), 502–515. 10.1080/00325481.2016.1188319 27166559

[CIT0058] Treede, R.D., Rief, W., Barke, A., Aziz, Q., Bennett, M.I., Benoliel, R. et al., 2019, ‘Chronic pain as a symptom or a disease: The IASP Classification of Chronic Pain for the International Classification of Diseases (ICD-11)’, *Pain* 160(1), 19–27. 10.1097/j.pain.0000000000001384 30586067

[CIT0059] Visagie, S. & Schneider, M., 2014, ‘Implementation of the principles of primary health care in a rural area of South Africa’, *African Journal of Primary Health Care and Family Medicine* 6(1), 1–10. 10.4102/phcfm.v6i1.562 PMC4502891 26245391

[CIT0060] Vos, T., Lim, S.S., Abbafati, C., Abbas, K.M., Abbasi, M., Abbasifard, M. et al., 2020, ‘Global burden of 369 diseases and injuries in 204 countries and territories, 1990–2019: A systematic analysis for the Global Burden of Disease Study 2019’, *The Lancet* 396(10258), 1204–1222. 10.1016/S0140-6736(20)30925-9 PMC7567026 33069326

[CIT0061] Wilson, S., Chaloner, N., Osborn, M. & Gauntlett-Gilbert, J., 2017, ‘Psychologically informed physiotherapy for chronic pain: Patient experiences of treatment and therapeutic process’, *Physiotherapy* 103(1), 98–105. 10.1016/j.physio.2015.11.005 27095482

[CIT0062] World Medical Association, 2013, ‘World Medical Association Declaration of Helsinki: Ethical principles for medical research involving human subjects’, *Journal of American Medical Association* 310(20), 2191–2194. 10.1001/jama.2013.281053 24141714

